# Biological Activities of Essential Oils and Hydrolates from Different Parts of Croatian Sea Fennel (*Crithmum maritimum* L.)

**DOI:** 10.3390/biom15050666

**Published:** 2025-05-04

**Authors:** Livia Slišković, Nikolina Režić Mužinić, Olivera Politeo, Petra Brzović, Josip Tomaš, Ivana Generalić Mekinić, Marijana Popović

**Affiliations:** 1 University Department for Forensic Sciences, University of Split, R. Boškovića 33, 21000 Split, Croatia; lsliskovic@forenzika.unist.hr; 2 Department of Medical Chemistry and Biochemistry, School of Medicine, University of Split, Šoltanska 2a, 21000 Split, Croatia; nikolina.rezic.muzinic@mefst.hr; 3 Department of Biochemistry, Faculty of Chemistry and Technology, University of Split, R. Boškovića 35, 21000 Split, Croatia; olivera@ktf-split.hr; 4 Department of Food Technology and Biotechnology, Faculty of Chemistry and Technology, University of Split, R. Boškovića 35, 21000 Split, Croatia; petra.brzovic@ktf-split.hr (P.B.); jtomas@unist.hr (J.T.); 5 Department of Applied Science, Institute for Adriatic Crops and Karst Reclamation, Put Duilova 11, 21000 Split, Croatia

**Keywords:** *Crithmum maritimum* L., essential oils, hydrolates, GC-MS, HS-SPME, antioxidant activities, acetylcholine inhibitory activity, butyrylcholine inhibitory activity, cytotoxic activities, multivariate analysis

## Abstract

The traditional nutritional use of sea fennel (*Crithmum maritimum* L.) has been rediscovered and reestablished, making this halophyte plant a prominent ingredient in coastal cuisine and a subject of interest in various scientific disciplines, including pharmacy and medicine. The first objective of this study was to identify the volatile profiles of essential oils (EOs) and hydrolates derived from the leaves, flowers, and fruits of sea fennel using gas chromatography–mass spectrometry. A total of 25 different volatiles were identified in the EOs and 63 were identified in the hydrolates. Limonene was the most abundant component in the EOs (74.85%, 74.30%, and 67.41%, respectively), while in the hydrolates, it was terpinen-4-ol in the leaves (27.8%) and the flowers (36.7%) and (*Z*)-carveol in the fruits (11.4%). The second objective was to investigate the biological activities of the samples. The antioxidant and choline inhibitory activities of hydrolates were generally low, with the flower hydrolate providing the inhibition of both enzymes and the leaf hydrolate with the highest antiradical activity. The cytotoxic activities of the EOs and hydrolates were also investigated. The human breast adenocarcinoma cell line MDA-MB-23 was the most sensitive against EOs from the flowers and fruits, reaching the IC_50_ after 48 and 72 h, respectively. The leaf hydrolate exhibited cytotoxic activity after 72 h, while the flower hydrolate was effective after 48 h. The MCF-7 cell line was sensitive to the flower and fruit EOs, and the IC_50_ was reached at all the tested periods. Overall, the results highlight sea fennel as a rich source of bioactive compounds that have significant potential for greater utilization in the nutraceutical and pharmaceutical industries.

## 1. Introduction

The increasing salinization of soils and waters due to climate change is driving interest in salt-tolerant crops like sea fennel. This emerging halophyte, with an exceptional resilience to harsh environmental conditions, has developed unique adaptations to saline environments, including the production of biologically active compounds that boost both its survival and nutritional profile [1,2]. Due to its potential as a “cash crop”, sea fennel is gaining increasing attention as a sustainable agricultural option in coastal regions affected by soil deterioration and water pollution [3,4,5].

The use of sea fennel (*Crithmum maritimum* L.) dates back to ancient times, when coastal communities consumed it mainly out of necessity. Sea fennel leaves were particularly valued for their role in human nutrition, as they are rich in vitamin C and were used to prevent scurvy, which was a significant concern among sailors and maritime populations [6,7]. Today, however, it has been rediscovered and has rightfully earned its status as a star of coastal cuisine, but its application is also expanding into other sectors, such as cosmetics, food supplements, and the pharmaceutical industry [8]. The aerial parts of sea fennel contain a wealth of bioactive compounds, including essential oils (EOs), vitamins C and E, phenolics, carotenoids and essential fatty acids, essential amino acids, dietary fibers, and minerals, especially iodine. The distribution of metabolites varies among the different plant parts (leaves, flowers, stems, and fruits) [7,9,10,11,12].

The most significant difference among the sea fennel populations in the Mediterranean basin lies in the composition of their essential oils. The key distinguishing factor is the presence of the volatile compound dillapiole [13]. The Croatian sea fennel chemotype is completely free of dillapiole, which has been confirmed by various studies that have analyzed different parts of the plant, collected from various locations along the Adriatic coast and over different time periods [10,14,15].

Nowadays, we are witnessing a growing consumer demand for natural products and their derivatives. Due to their richness in bioactive compounds, essential oils (EOs) are increasingly being investigated for their potential as preservatives, flavorings, and therapeutic agents [16,17]. Hydrolates (aromatic waters, hydrosols) are another highly valued natural product obtained as a by-product of the EO distillation process, and they are increasingly attracting interest in fields such as aromatherapy, perfumery, and food flavoring [18,19]. Hydrolates are highly diluted mixtures of volatile compounds generated during the distillation and condensation of the oil and water vapors. Due to the immiscibility and differences in the densities of these two components, essential oils separate into the upper layer, while hydrolates, being denser, form the lower layer [20]. However, polar volatiles from EOs, mainly oxygenated compounds that are capable of forming hydrogen bonds, often migrate from the EOs into hydrolates, contributing to their aroma and properties. This is often the reason why the chemistry of EOs differs from that of their respective hydrolates [21,22]. In addition to the chemistry of EOs and hydrolates, the bioactivity assessments of these fractions, including their antioxidant, antimicrobial, and anti-inflammatory properties, have attracted the interest of scientists over the last few decades, especially due to their more frequent application in food/pharmaceutical/cosmetic products [23,24,25,26].

Although the EOs from sea fennel have been well characterized, there is little or no information on the corresponding hydrolates [21,22].

Due to the increasing demand for natural products and their wide range of applications, this study conducted a comprehensive chemical and biological analysis of sea fennel essential oils and hydrolates. To the best of our knowledge, the chemical composition of hydrolates derived from sea fennel flowers and fruits has not been previously reported. Likewise, no existing literature was found on the cytotoxic effects of sea fennel EOs or hydrolates against the MDA-MB-231, MCF-7, or OVCAR-3 cell lines, or on the acetylcholinesterase (AChE) and butyrylcholinesterase (BuChE) inhibitory activities of sea fennel hydrolates. To expand the knowledge on the potential health benefits of sea fennel, the authors investigated the volatile profiles of the leaf, flower, and fruit EOs and hydrolates, along with their antioxidant, cholinesterase-inhibitory, and cytotoxic effects.

## 2. Materials and Methods

### 2.1. Plant Material

Sea fennel (*Crithmum maritimum* L.) aerial parts were collected from the same location and increments in June (leaves), August (flowers), and November (fruits) 2023 in Split, Croatia (43.5147° N, 16.4435° E). The plant material was air-dried for two weeks at room temperature in a shaded and aerated place, and later used for the isolation of EOs. A voucher specimen of plant material was deposed in an herbarium at the Faculty of Chemistry and Technology (University of Split, Split, Croatia).

### 2.2. Extraction

#### 2.2.1. Hydrodistillation of EOs

The EOs and hydrolates of sea fennel were extracted from 100 g of plant material by hydrodistillation (HD) in a modified Clevenger-type apparatus, as detailed by Popović et al. [27]. The distillation lasted 3 h and a mixture of pentane/diethyl ether (2:1, *v*/*v*) was used as a solvent trap for the EO’s lipophilic components. The extracted EOs were dried over anhydrous sodium sulphate (Kemika, Zagreb, Croatia) and stored at −20 °C until further analysis. Likewise, the hydrolates, the water layer under the solvent trap, were separated and stored at −20 °C for subsequent analysis, as described by Politeo et al. [22].

#### 2.2.2. Isolation of VOCs from Hydrolates

The isolation of VOCs from hydrolates was performed according to Politeo et al. [22] by solid-phase microextraction (SPME) fibers with a 50/30 µm DVB/CAR/PDMS coating purchased from Supelco (Sigma Aldrich, Bellefonte, PA, USA). Before use, the fibers were conditioned at 250 °C for 1 h. For sample preparation, the hydrolates and NaCl were placed in 20 mL glass vials, which were then sealed using magnetic caps with silicone septa. The SPME fibers were inserted into the vial headspace, and the samples were equilibrated at 40 °C for 30 min. Subsequently, the fibers were transferred to the GC injection port for thermal desorption, which was conducted at 250 °C for 3 min. Each sample analysis was performed in duplicate.

### 2.3. Identification of the Volatiles from EOs and Hydrolates by GC-MS

The identification and quantification of volatiles in the EOs and hydrolates of sea fennel were conducted using GC-MS. A gas chromatograph (model 8890 GC) equipped with an automatic liquid injector (model 7693A) and a tandem mass spectrometer (MS, model 7000D GC/TQ, Agilent Inc., Santa Clara, CA, USA) were used for the analysis. A non-polar HP-5MS UI column (30 m × 0.25 mm × 0.25 µm, Agilent Inc., Santa Clara, CA, USA) was used for chromatographic separation. Helium served as the carrier gas with a flow rate of 1.0 mL/min, with a sample injection volume of 1 µL. The analyses were performed in MS full-scan mode (33–350 *m*/*z*), the ion source temperature was set to 200 °C, the interface temperature to 250 °C, and the ionization voltage to 70 eV.

For the EO analysis, the column temperature was held at 60 °C for 3 min and then increased at a rate of 3 °C/min to 246 °C, where it was maintained isothermally for 25 min. For the hydrolate analysis, the oven temperature was initially held at 40 °C for 3 min and then increased to 80 °C at 3 °C/min, followed by a further increase to 220 °C at a rate of 10 °C/min, where it was held for 5 min.

Compound identification was based on comparing the retention indices with a series of *n*-hydrocarbons and matching the mass spectra with commercial databases (Wiley 7 MS library, Wiley, NY, USA, and NIST02, Gaithersburg, MD, USA) and the literature [28]. Quantification was performed by calculating the relative peak areas (%) using the normalization method (without correction factors). All the analyses were conducted in duplicate, and the percentages shown in Table 1 and Table 2 represent the mean values.

### 2.4. Antioxidant Activities

The antioxidant assays were performed in 96-well plates using a Tecan MicroPlate Reader, model Sunrise (Tecan Group Ltd., Männedorf, Switzerland), and a Synergy HTX Multi-Mode Reader (BioTek Instruments, Inc., Winooski, VT, USA). All the measurements were carried out in triplicate.

The reducing activity of the samples was evaluated using the Ferric reducing antioxidant power (FRAP) assay, following the protocol by Čagalj et al. [29]. The procedure involves adding samples (10 µL) to a FRAP reagent mixture (300 µL, acetate buffer (0.3 M)/TPTZ (10 mM) in HCl (40 mM)/FeCl_3_ (20 mM) = 10:1:1) and measuring the absorbance at 593 nm after 4 min. The results were compared with a calibration curve prepared using different concentrations of Fe^2+^ and expressed as µM of Fe^2+^.

An oxygen radical absorbance capacity (ORAC) assay was conducted as described by Čagalj et al. [29]. The reagent mixture of fluorescein (150 μL, 0.08 mM in 0.075 M phosphate buffer) and the sample (25 μL) (or Trolox for the calibration curve/buffer for blank measurements) was added into microplate wells and thermostated (30 min at 37 °C). The reaction, initiated by adding 2,2’-azobis(2-methylpropionamidine) dihydrochloride (25 µL), was monitored for 80 min at the excitation and emission wavelengths of 485 and 520 nm, with measurements taken every minute. The results are expressed in micromolar Trolox equivalents (µM TE), calculated from the calibration curve obtained by testing different concentrations of Trolox.

The 2,2-diphenyl-1-picrylhydrazyl (DPPH) radical scavenging activity of the samples was assessed by following the method of Čagalj et al. [29]. In this assay, the decrease in absorbance of a DPPH ethanolic solution (290 µL, initial absorbance of 1.2 ± 0.02) after the addition of the sample (10 µL) was measured at 517 nm after 1 h. The results are expressed as the percentage inhibition of the DPPH radical (inhibition %), calculated according to the following equation:

Inhibition % = [(Ac − As)/Ac] × 100,

where Ac is the initial absorbance of the DPPH solution and As is the absorbance of the DPPH solution containing the sample 1 h after its addition.

### 2.5. Cholinesterase Inhibitory Activity

The samples’ inhibition capacity of AChE and BuChE was examined by modified Ellman’s assays, as described by Bektašević et al. [30]. The reaction mixture consisted of phosphate buffer (0.1 M, pH = 8), 10 µL of DTNB (2-nitro-5-thiobenzoate, 6.6 mM, dissolved in phosphate buffer, pH = 7), 10 µL of the sample solution or distilled water as a negative control, and 10 µL of enzyme solutions (AChE, BuChE, 0.66 U/mL). Acetylthiocholine (ATChI) or butyrylthiocholine iodide (BTChI) (10 µL, 11 mM, prepared in phosphate buffer, pH = 8), was used to initialize the reaction. Non-enzymatic hydrolysis was also monitored by measuring blank runs. All the spectrophotometric measurements were carried out at 405 nm during 6 min and performed in quadruplicate. Finally, the inhibition, expressed as a percentage, was calculated by the following formula:

Inhibition (%) = ((Ae − Abe) − (Au − Abu))/((Ae − Abe)) × 100,

where Ae represents the absorbance of the enzyme solution without the sample (inhibitor), Abe is the absorbance of the blank sample of the enzyme solution without the enzyme, Au is the absorbance of the enzyme solution with the sample (inhibitor), and Abu is the absorbance of the blank sample of the enzyme solution without the sample (inhibitor).

### 2.6. Cytotoxic Activities

The cytotoxic activity of sea fennel EOs and hydrolates was determined by a cell viability assay (gold standard) using 3-(4,5-dimethylthiazol-2-yl)-2,5-diphenyltetrazolium bromide (MTT). The MTT assay was performed on four cell lines: the human breast adenocarcinoma MDA-MB-231 cell line, the human breast metastatic adenocarcinoma MCF-7 cell line, the human ovarian carcinoma OVCAR-3 cell line (LGC standards), and the human embryonic kidney HEK-293 cell line (ATCC Cell Lines), according to Režić Mužinić et al. [31]. The MDA-MB-231, MCF-7, OVCAR-3, and HEK-293 cell lines were seeded in 96-well plates at a density of 9500, 7000, 8000, and 9000 cells/well, respectively, and incubated overnight for adherence. The cells were then treated with the medium or serial dilutions of extracts (1–250 µg/mL) for 4, 24, 48, and 72 h (in triplicate). After treatment, 100 µL of a 0.5 g/L MTT solution was added, and the cells were incubated at 37 °C for 2 h. MTT was replaced with 10% DMSO to dissolve the formazan crystals for ten minutes. Purpuric-colored products were measured at 570 nm using a microplate reader (BioSan, Riga, Latvia). The IC_50_ values were calculated in the IC_50_ calculator (AAT Bioquest, Sunnyvale, CA, USA).

### 2.7. Statistical Analysis

#### 2.7.1. ANOVA

To determine the percentage differences in the volatile compounds of sea fennel EOs and hydrolates from the leaves, flowers, and fruits, a one-way ANOVA with a Dunnett T3 post hoc significance test for unequal variances was performed at the significance level of *p* ≤ 0.05 by the SPSS software, version 25.0, IBM Corporation, New York, NY, USA.

Additionally, to evaluate the differences in the concentrations of sea fennel EOs from the leaves, flowers, and fruits on different cancer cell lines, a one-way ANOVA was performed by the Past 3.X software (version 3.14, University of Oslo, Oslo, Norway), with the significance level at *p* < 0.05.

#### 2.7.2. Linear Regression

To determine the impact of various factors on the cytotoxic activity of the essential oils and hydrolates from sea fennel leaves, flowers, and fruits, a linear regression analysis was conducted using the SPSS software, version 25.0 (IBM Corporation, New York, NY, USA). The dependent variable in the analysis was the IC_50_ value, while the independent variables included the volatile compounds, essential oil concentration, plant part, and exposure time (4, 24, 48, and 72 h). The aim of the analysis was to determine the most significant predictors of cytotoxicity. Prior to the regression analysis, the dataset was checked for linearity, normality, and multicollinearity to ensure the validity of the model.

## 3. Results and Discussion

### 3.1. Volatile Composition of Sea Fennel Leaf, Flower, and Fruit EOs and Hydrolates

**Figure 1:**
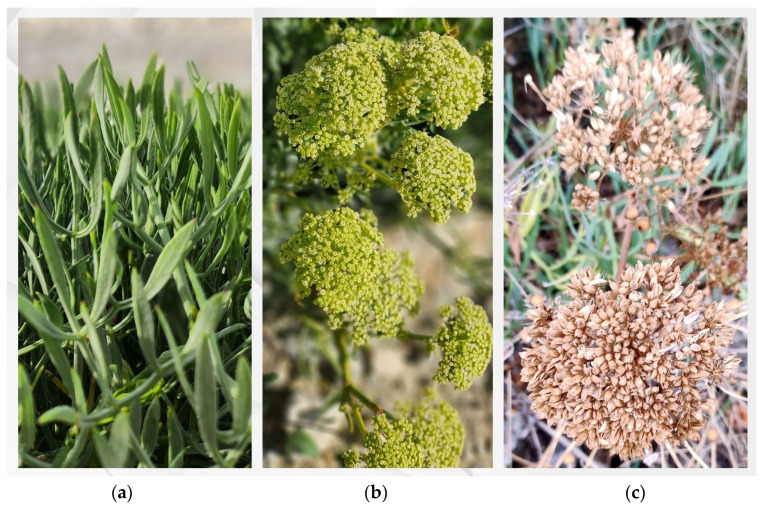
Sea fennel (**a**) leaves, (**b**) flowers, and (**c**) fruits.

EOs from sea fennel leaves, flowers, and fruits (Figure 1) were obtained by hydrodistillation and analyzed by GC-MS (Table 1), along with their corresponding hydrolates (Table 2). While the EOs from sea fennel leaves and flowers of the Croatian chemotype have been extensively studied and well documented [10,14,15], there have been no reports focusing on the chemistry of the fruit EO. Likewise, while some studies have investigated hydrolates from sea fennel leaves [18,19], this study is, to the best of the authors’ knowledge, the first to report the volatile composition of hydrolates obtained from other plant parts.

The classification of sea fennel chemotypes remains a topic of debate. Pateira et al. [32] identified two chemotypes distinguished by their dillapiole content: chemotype I contains 15–17%, while chemotype II has 0–6%. In contrast, due to significant variations in essential oil composition depending on the geographical origin, Renna [33] proposed a broader classification consisting of four chemotypes: the aromatic monoterpene type, the monoterpene hydrocarbon type, the phenylpropanoid type, and an intermediate type.

Previous studies on Croatian sea fennel [10,14,15] have classified its EOs as chemotype II and of the monoterpene hydrocarbon type. The most abundant compounds in all the plant parts were monoterpene hydrocarbons, reaching 97.87% in the flowers, 96.76% in the fruits, and 85.01% in the leaves (Table 1). The GC-MS analysis of sea fennel leaf, flower, and fruit EOs conducted in this study supports this classification, as dillapiole was completely absent, while limonene (a monoterpene hydrocarbon) was the predominant compound, ranging from 68.41% in the fruits to 74.85% in the leaves. Sabinene, another abundant monoterpene hydrocarbon, was absent in the leaf essential oil, but reached 15.72% in the flowers and 12.37% in the fruits. The limonene content in the leaf (74.85%) and flower (74.30%) EOs was consistent with the literature data, according to which the EOs from summer leaves from the Split region contained limonene levels ranging from 57.5 [15] to 79.13% [10] and up to 84.53% [27]. In flower EOs, limonene ranged from 62.2 [15] to as high as 93.39% (based on our unpublished data). Previous studies have reported sabinene levels in leaves ranging from 0.15 [27] to 51.47% [21], and in flowers from 1.65 (unpublished data) to 12% [15]. Additionally, two other monoterpene hydrocarbons were found in notable amounts: *α*-pinene was the most abundant in the fruit EO (9.07%), while *β*-ocimene was the highest in the leaves (6.54%), slightly exceeding previously published values (0.42–4.1%) [10,21]. To evaluate the differences among EOs from different plant parts, a one-way ANOVA was performed. The level of *α*-pinene showed significant differences across all the samples. Although there was no significant variation in the limonene content between the leaf and flower essential oils, a notable difference was observed between the fruit and leaf essential oils. In contrast, the *β*-ocimene levels differed significantly between the leaf and flower essential oils, as well as between the leaf and fruit essential oils.

The most abundant compounds in sea fennel EOs are of significant value to human health. Limonene, an aromatic and volatile monoterpene, exhibits a wide range of biological activities, including anticancer, antidiabetic, gastroprotective, anti-inflammatory, antioxidant, antinociceptive, antihyperalgesic, and antiviral properties [34]. Another notable compound, *α*-pinene, has been shown to possess cytoprotective, gastroprotective, neuroprotective, anxiolytic, and anticonvulsant effects [35]. Although sabinene has been less extensively researched, an available study suggest that it exhibits angiostatic and antiangiogenic effects, as well as antimicrobial activity [36].

To examine the differences in sea fennel leaf, flower, and fruit hydrolates, an HS-SPME-GC-MS analysis was performed (Table 2). As expected, the hydrolates contained more polar compounds than those found in the EOs, since their constituents are generally water-soluble. Monoterpenes, the predominant constituents of the EOs, were typically absent in the hydrolates due to their higher affinity for lipophilic environments [37]. In total, 63 volatile compounds were identified in the sea fennel hydrolates. The greatest diversity was observed in the fruit hydrolate, with 48 compounds identified, followed by the leaf hydrolate, with 43, and the flower hydrolate, with 32 compounds. Monoterpenoids represented the most abundant class of compounds across all three plant parts, with the highest proportion observed in the fruits (87.4%) and the lowest in the leaves (75.25%). The second most prevalent group was monoterpene hydrocarbons, accounting for 12.55% in the leaves, while sesquiterpenoids were notably present in the flowers (10.7%). Ketones, esters, and phenol were detected only in the leaves (Table 2). Considering that the volatiles in the hydrosols predominantly originated from oxygenated constituents of the essential oils, the high content of monoterpenoids in the hydrosols was an expected outcome.

Terpinen-4-ol was identified as the most abundant compound in the leaf and flower hydrolates (27.8% and 36.7%, respectively) and was also present in significant amounts in the fruit hydrolate (10.6%). Sabinene, one of the dominant compounds in the flower and fruit EOs, appeared in the hydrolates in its oxygenated forms, (*Z*)- and (*E*)-sabinene hydrate. (*Z*)-Sabinene hydrate was detected at 5.9% in the flowers and 2.5% in the fruits, while (*E*)-sabinene hydrate was found at 7.4% in the flowers and 3.0% in the fruits. Similarly, (*Z*)- and (*E*)-carveol, the hydroxylated derivatives of limonene, were detected in high concentrations across all samples. (*Z*)-Carveol was most abundant in the fruit hydrolate (11.4%), while (*E*)-carveol was the highest in the leaf hydrolate (7.3%). The fruit hydrolate was also rich in (*E*)-isocarveol (10.6%) and (*E*)-*p*-mentha-2,8-dien-1-ol (9.0%). The flower hydrolate contained a considerable amount of spathulenol (8.3%), while the leaf hydrolate was characterized by 10-(acetylmethyl)-3-carene (8.6%), which was absent in the flower and fruit hydrolates. Previous studies on sea fennel leaf hydrolates collected in Central Dalmatia in August and October similarly identified terpinen-4-ol as the most abundant volatile compound (13.86% and 41.93%, respectively), followed by 10-(acetylmethyl)-3-carene (13.45% and 13.80%, respectively). Comparable levels of sabinene hydrates (1.54–4.73%) and (*E*)-carveol (3.36%–4.92%) were also reported [21,22]. α-Terpineol was present in similar concentrations in all the samples, and increased slightly from the leaves to the flowers to the fruits (4.2% < 5.8% < 6.9%). A one-way ANOVA revealed no significant differences in the *α*-terpineol levels between the samples. However, significant differences were found for terpinen-4-ol, (*Z*)-carveol, and carvone in all the hydrolates. The flower hydrolate exhibited significantly different levels of (*Z*)- and (*E*)-sabinene hydrate and spathulenol compared to the other hydrolates, while the fruit hydrolate differed significantly in its content of (*E*)-*p*-mentha-2,8-dien-1-ol.

Terpinen-4-ol, the most abundant volatile compound in all the sea fennel hydrolates, exhibits a wide range of pharmacological properties, including strong antimicrobial and antivirulent activity as well as antioxidant, anti-inflammatory, antihypertensive, and anticancer effects. Isolated mainly from tea tree oil, terpinen-4-ol can be synthesized by the hydration of sabinene or obtained via the oxidation, hydrogenation, and selective hydrogenation of terpinolene [38]. Terpineol occurs in four isomeric monoterpenoid forms, including terpine-4-ol and α-terpineol. Due to its pleasant fragrance, it is widely used in the perfume and cosmetics industry. Additionally, it has shown a range of biological activities, such as antioxidant, anticancer, antihypertensive, anticonvulsant, antiulcer, and antinociceptive properties [39]. Carvone and carveol, known for their aromatic properties, are extensively used in the flavor and fragrance industry. However, their potential extends beyond these applications, as they also exhibit antioxidant, antimicrobial, anti-inflammatory, antidiabetic, antitumor, and insecticidal activities [40]. Spathulenol, a tricyclic sesquiterpene alcohol, possesses antioxidant, antiseptic, immunomodulatory, and antinociceptive properties. Its potential use for the treatment of cardiac fibrosis, as an adjunct in chemotherapy, and in the therapy of neurodegenerative diseases have also been explored [41].

### 3.2. Biological Activities of Sea Fennel Leaf, Flower, and Fruit EOs and Hydrolates

Sea fennel extracts have been extensively studied for their bioactive constituents and properties, among which the antioxidant activity has attracted the most interest. Previous studies on Croatian sea fennel populations have confirmed the good antioxidant activity of the non-volatile fractions (polar/phenolic extracts) [14,15], while the activity of the tested EOs was extremely low [15,42] due to a high content of monoterpenic hydrocarbons that are known as weak radical scavengers and reducing agents. Therefore, in order to provide novelty in the research, this study focused solely on the antioxidant properties of the corresponding hydrolates (from different plant parts, Table 3), which were characterized by the presence of polar, water-soluble, and, in most cases, oxygenated compounds. To the best of the authors’ knowledge, this is first report to compare the antioxidant potential of hydrolates from different parts of the sea fennel plant, namely flowers, leaves, and fruits.

As can be seen from the results shown in Table 3, the reducing activity of the samples, assessed using the FRAP method (which measures the ability of antioxidants to reduce Fe^3+^ to Fe^2+^ under acidic conditions), was not observed. This finding aligns with our previous study [21], where the reducing activity was negligible compared to more potent antioxidants. The DPPH and ORAC method, on the other hand, provided information about the free radical scavenging activity of the samples. The difference was in the fact that the DPPH uses stabile, synthetic radicals, whereas the ORAC method monitors the inhibition of peroxyl radicals, which are common in biological systems. Based on the obtained results, it can been seen that the DPPH method also did not show significant activity, with inhibition percentages ranging from 5.67 for the fruit hydrolate to 6.30 for the leaf hydrolate, respectively. However, for the ORAC method, slight differences were seen between the tested results. The highest activity was detected for the leaf hydrolate (123 µmTE/L), which was close to previously reported values [21]. The relative weak antioxidant activity of the fruit hydrolate can be potentially attributed to this hydrolate having the lowest amount of terpinen-4-ol compared to the other samples, while the leaf hydrolate had higher levels of (*E*)-carveol and carvone, as well as 10-(acetylmethyl)-3-carene (8.6%), which was not detected in the other two samples.

Similar to the antioxidant activity, the enzyme inhibitory activity of sea fennel EOs has been previously studied by various authors [15,43,44,45,46,47], but there is limited information on the activity of sea fennel hydrolates. In our previous study, we tested the antityrosinase and anticollegenase inhibitory activity of sea fennel hydrolates [21], whereas this research focused on their activity on AChE and BuChE. The obtained results are presented in Table 3.

Although several studies have investigated the AChE and/or BuChE inhibitory activity of sea fennel’s polar, non-volatile fractions/extracts [44,48], only two studies, including one from our research group, have investigated the inhibitory activity of EOs [15,47]. To the authors’ knowledge, no study has examined the AChE and/or BuChE activity of hydrolates.

Ismail et al. [47] investigated the enzyme inhibition activities of a sea fennel EO (from the coastal region of Libya, North Africa) on AChE and detected an IC_50_ value of 34.43 mg/mL. However, their EO was characterized by a different volatile profile, with the major constituents being thymyl methyl ether (56.86%), *γ*-terpinene (16.17%), and ledene oxide (4.32%). In our previous study, EOs from sea fennel flowers and leaves were tested at a concentration of 1 mg/mL, and the IC_50_ values were not detected. However, according to the obtained results, the flower EO was more effective at inhibiting AChE, while the leaf EO provided greater inhibition against BuChE. The EOs from flowers and leaves exerted better inhibition against AChE rather than BuChE [15]. The results for the AChE and BuChE inhibitory activity of hydrolates are presented in Table 3, and they also indicate better activity of the flower hydrolate against AChE than BuChE (two-fold lower inhibition). This sample was recognized for the highest content of terpinen-4-ol, which could be the reason for its inhibition activity, as previously reported. The activity of the hydrolates on the inhibition of AChE and BuChe could be explained by terpinene-4-ol, 1,8-cineole, and linalool. In the case of the leaf hydrolate, activity only against BuChE was recorded (3.31% inhibition).

**Figure 2:**
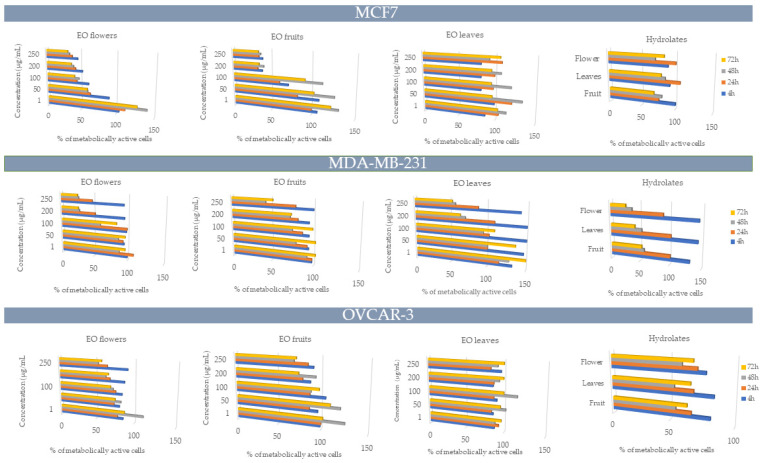
Percentage of metabolically active human breast adenocarcinoma MDA-MB-231, human breast metastatic adenocarcinoma MCF-7, and human ovarian carcinoma OVCAR-3 cell lines after 4, 24, 48, and 72 h of incubation with different concentrations of sea fennel essential oils and hydrolates from leaves, flowers, and fruits.

The cytotoxic effects of sea fennel EOs from the leaves, flowers, and fruits at different concentrations, along with the hydrolates, were tested against three human cancer cell lines (MDA-MB-231, MCF-7, and OVCAR-3) after 4, 24, 48, and 72 h of incubation. The IC_50_ values are presented in Figure 2. Additionally, healthy human embryonic kidney HEK-293 cells were treated with fruit and flower EOs, and the results are shown in Figure S3.

Of the three tested cell lines, cytotoxic activity was observed against two, namely MDA-MB-231 and MCF-7, while none of the EOs or hydrolates reached the IC_50_ value for any tested concentration on the OVCAR-3 cell line (Figure 2). The human breast adenocarcinoma MDA-MB-231 cell line was the most sensitive to the sea fennel flower EO at concentrations of 200 and 250 μg/mL, with 28.25 and 27.36% cell survival after 48 h and 26.46% and 25.71% cell survival after 72 h, respectively. The fruit EO reached the IC_50_ (250 μg/mL) after 48 h with 43.39% cell survival. The flower hydrolate reached the IC_50_ after 48 h (with 37.42% cell survival) and after 72 h (with 26.75% cell survival), while the leaf hydrolate reached the IC_50_ (with 41.92% cell survival) only after 72 h. The human breast metastatic adenocarcinoma MCF-7 cell line was sensitive to flower and fruit EOs for all the tested times, but the best results were observed after 72 h of incubation (IC_50_ = 250 μg/mL), with 31.97% and 34.22% cell survival.

No reduction in the amount of healthy human embryonic kidney HEK-293 cells (Figure S3) was observed when they were treated with different concentrations of fruit and flower essential oils (the EOs that had the highest cytotoxic activity on cancer cell lines). Interestingly, at concentrations <200 μg/mL, the cell count doubled after 48 and 72 h, while at a concentration of 250 μg/mL, some cytotoxic effects were observed, but the IC_50_ was not reached. These results suggest the need for further research on sea fennel EOs regarding the potential mechanism of their healing properties [49].

Since essential oils and hydrolates contain a wide range of diverse components, and their cytotoxic activity was tested at different concentrations and exposure times, a linear regression analysis was performed to evaluate the impact of various factors on the cytotoxic activity of EOs and hydrolates from sea fennel leaves, flowers, and fruits (Table 4 and Table 5). The concentration of the EOs had the greatest effect on the MCF-7 cell line (B = −0.226, *p* < 0.0001), with a lower IC_50_ value observed at higher concentrations. The concentration was a significant factor for all the cell lines tested. For the MCF-7 and MDA-MB-232 cell lines, octanal showed a significant effect (B = 75.86, *p* < 0.0001 and B = 74.10, *p* < 0.0001, respectively).

The only factor that had a significant effect on all the hydrolate-treated cell lines was time (Table 5). For all three cell lines (MCF-7, MDA-MB-232, and OVCAR-3), the coefficients were negative (B = −0.28, B = −1.51, and B = −0.254, respectively), indicating that the IC_50_ values decreased with increasing exposure time.

Regarding the chemistry of essential oils, it can be suggested that the high concentration of limonene plays a key role in the strong cytotoxic activity of sea fennel EOs. Limonene is well known for its chemopreventive properties against various types of cancer and possesses no mutagenic, carcinogenic, or nephrotoxic risks to humans [50]. However, the presence of α-pinene [51] and several other biologically active compounds suggests that the cytotoxic effects observed for sea fennel EOs may be due to their synergistic effect. Since hydrolates are highly diluted, they generally exhibit less biological activity than their corresponding essential oils. However, due to the presence of a wide variety of compounds with anticancer properties, such as terpinen-4-ol, α-terpineol, carvone, and carveol [38,39,40], as well as their probable synergistic effects, the sea fennel hydrolates also demonstrated cytotoxic activity.

Previous studies have investigated the cytotoxicity of sea fennel aqueous and chloroform extracts against tumor cell lines [52,53], and one study examined the cytotoxic effects of a sea fennel EO. Beeby et al. also performed studies on the MCF-7 and HER-293 cell lines; however, due to the dominance of *γ*-terpinene, thymol methyl ether, *o*-cymene, and *β*-phellandrene in their EO sample, compounds that were minor or absent in our EOs, a direct comparison of the results is not possible [54].

To the best of the authors’ knowledge, this is the first report on the cytotoxic activities of sea fennel essential oils (chemotype II, monoterpene hydrocarbon type) and hydrolates against the MDA-MB-231, MCF-7, and OVCAR-3 cell lines.

## 4. Conclusions

This study provides new insights into the chemical composition and biological activities of essential oils and hydrolates from different parts of sea fennel (*Crithmum maritimum* L.). The volatile profiles of EOs from the leaves, flowers, and fruits were dominated by monoterpene hydrocarbons, with limonene as the most abundant constituent (up to 74.85%). The hydrolates were more chemically diverse than the EOs and were dominated by monoterpenoids, with terpinen-4-ol found in the highest amount in the leaves (27.8%). The results demonstrate weak antioxidant and enzyme-inhibitory and significant cytotoxic activities, with variations depending on the plant part and extract type. The essential oils showed significant effects against certain cancer cell lines, while the hydrolates exhibited moderate activity, without any toxicity to healthy cells for all the tested extracts.

These findings highlight the potential of sea fennel extracts for applications in the nutraceutical and pharmaceutical industries, particularly due to their bioactive properties. Further investigations are needed to explore their mechanisms of action, safety profiles, and possible therapeutic applications.

## Figures and Tables

**Table 1:** GC-MS analysis of sea fennel leaf, flower, and fruit essential oils.

No.	Compound	LRI	Leaves	Flowers	Fruits	Mode of Identification
1.	Hexanal	837	0.70 ± 0.03	nd	nd	GC, MS
2.	Acetic acid ethyl ester	842	1.41 ± 0.08	nd	nd	GC, MS
3.	Heptanal	898	0.41 ± 0.01	nd	nd	GC, MS
4.	*α*-Pinene	927	3.20 ± 0.02 a	1.01 ± 0.02 b	9.07 ± 0.04 c	GC, MS
5.	Camphene	941	nd	nd	0.18 ± 0.01	GC, MS
6.	Sabinene	966	nd	15.72 ± 0.22 a	12.37 ± 0.06 a	GC, MS
7.	*β*-Pinene	969	nd	nd	0.74 ± 0.00	GC, MS
8.	*β*-Myrcene	985	0.42 ± 0.01 a	1.04 ± 0.00 b	1.18 ± 0.02 b	GC, MS
9.	Octanal	999	0.49 ± 0.032 a	nd	0.15 ± 0.01 a	GC, MS
10.	*α*-Terpinene	1011	nd	0.52 ± 0.04 a	0.20 ± 0.00 a	GC, MS
11.	*o*-Cymene	1018	nd	0.35 ± 0.01 a	0.14 ± 0.01 b	GC, MS
12.	Limonene	1022	74.85 ± 0.03 a	74.30 ± 0.39 ab	68.41 ± 0.06 b	GC, MS
13.	*β*-Ocimene	1032	6.54 ± 0.04 a	3.72 ± 0.04 b	3.96 ± 0.02 b	GC, MS
14.	*γ*-Terpinene	1052	nd	1.23 ± 0.05 a	0.51 ± 0.03 b	GC, MS
15.	(*Z*)-Sabinene hydrate	1060	nd	nd	0.30 ± 0.01	GC, MS
16.	(*E*)-Sabinene hydrate	1092	nd	nd	0.20 ± 0.00	GC, MS
17.	(*Z*)-*p*-Mentha-2,8-dien-1-ol	1114	1.32 ± 0.01	nd	nd	GC, MS
18.	(Z)-Limonene oxide	1127	nd	nd	0.23 ± 0.00	GC, MS
19.	(*E)*-Limonene oxide	1131	nd	nd	0.26 ± 0.00	GC, MS
20.	Terpinen-4-ol	1171	nd	1.56 ± 0.01 a	0.66 ± 0.00 b	GC, MS
21.	Dodecane	1181	nd	nd	nd	GC, MS
22.	α-Terpineol	1184	nd	nd	0.37 ± 0.00	GC, MS
23.	Dodecane	1200	1.07 ± 0.03 a	0.30 ± 0.02 a	0.31 ± 0.01 a	GC, MS
24.	Tetradecane	1400	4.28 ± 0.25 a	0.25 ± 0.00 a	nd	GC, MS
25.	Hexadecane	1600	4.11 ± 0.08	nd	nd	GC, MS
*Aldehydes*			1.60	0.00	0.15	
*Esters*			1.41	0.00	0.00	
*Monoterpene hydrocarbons*			85.01	97.89	96.76	
*Monoterpenoids*			1.32	1.56	20.02	
*Alkanes*			9.46	0.55	0.31	
**Total chromatogram identified (%)**			98.80	100	99.24	

LRI—linear retention indices, nd—not detected. Identified compounds are expressed as mean ± SD. Different letters (a–c) denote statistically significant difference (*p* < 0.05).

**Table 2:** HS-SPME-GC-MS analysis of sea fennel leaf, flower, and fruit hydrolates.

No.	Compound	LRI	Leaves	Flowers	Fruits	Mode of Identification
1	α-Thujene	926	0.2 ± 0.00 a	nd	0.2 ± 0.05 a	GC, MS
2	(*E*)-2-Heptenal	949	nd	nd	0.1 ± 0.00	GC, MS
3	Benzaldehyde	952	1.00 ± 0.00 a	0.7 ± 0.00 b	0.1 ± 0.05 c	GC, MS
4	Sabinene	971	0.2 ± 0.00 a	0.3 ± 0.00 a	0.2 ± 0.00 a	GC, MS
5	*α*-Phellandrene	1001	1.00 ± 0.00 a	0.8 ± 0.00 b	1.1 ± 0.05 a	GC, MS
6	*α*-Terpinene	1012	0.45 ± 0.05 a	0.7 ± 0.00 b	0.4 ± 0.00 a	GC, MS
7	*p*-Cymene	1021	0.55 ± 0.05 a	nd	1.3 ± 0.05 b	GC, MS
8	*ß*-Phellandrene	1025	0.85 ± 0.05 a	1 ± 0.05 a	0.4 ± 0.00 b	GC, MS
9	1,8-Cineole	1027	0.5 ± 0.4 a	nd	0.3 ± 0.1 a	GC, MS
10	Benzeneacetaldehyde	1040	0.2 ± 0.00 a	0.9 ± 0.05 b	0.1 ± 0.15 a	GC, MS
11	γ-Terpinene	1057	0.35 ± 0.05 a	0.6 ± 0.00 b	0.4 ± 0.00 a	GC, MS
12	(*Z*)-Sabinene hydrate	1067	2.45 ± 0.05 a	5.9 ± 0.15 b	2.5 ± 0.3 a	GC, MS
13	*p*-Cymenene	1090	0.35 ± 0.05 a	nd	0.2 ± 0.05 b	GC, MS
14	Methyl benzoate	1092	0.25 ± 0.05	nd	nd	GC, MS
15	(*E*)-Sabinene hydrate	1096	3.15 ± 0.05 a	7.4 ± 0.15 b	3 ± 0.15 a	GC, MS
16	Linalool	1098	0.6 ± 0.00 a	1.2 ± 0.00 a	4.6 ± 2.15 a	GC, MS
17	(*E*)-*p*-Mentha-2,8-dien-1-ol	1120	4.8 ± 0.00 a	5.1 ± 0.05 a	9 ± 0.05 b	GC, MS
18	α-Campholenal	1124	nd	nd	0.1 ± 0.05	GC, MS
19	Cosmene	1130	nd	nd	0.6 ± 0.00	GC, MS
20	(*Z*)-*p*-Mentha-2,8-dien-1-ol	1135	1.7 ± 0.00 a	1 ± 0.00 b	5.1 ± 0.25 c	GC, MS
21	(*E*)-Limonene oxide	1136	0.7 ± 0.00	nd	nd	GC, MS
22	(*Z*)-*p*-Menth-2-en-1-ol	1140	2.3 ± 0.00 a	2.7 ± 0.05 a	0.6 ± 0.05 b	GC, MS
23	Camphor	1141	nd	nd	0.1 ± 0.00	GC, MS
24	(*E*)-Verbenol	1145	0.25 ± 0.05 a	nd	0.3 ± 0.05 a	GC, MS
25	Sabina ketone	1157	0.5 ± 0.00 a	0.5 ± 0.00 a	0.4 ± 0.1 a	GC, MS
26	Borneol	1165	nd	0.4 ± 0.1 a	0.3 ± 0.00 a	GC, MS
27	(*Z*)-Chrysanthenol	1168	nd	nd	0.8 ± 0.1	GC, MS
28	Terpinen-4-ol	1178	27.8 ± 0.1 a	36.7 ± 0.1 b	10.3 ± 1.5 c	GC, MS
29	*p*-Methyl acetophenone	1183	0.4 ± 0.00	nd	nd	GC, MS
30	*p*-Cymen-8-ol	1184	0.4 ± 0.1	nd	nd	GC, MS
31	Cryptone	1185	nd	nd	0.3 ± 0.05	GC, MS
32	(*E*)-Isocarveol	1188	0.65 ± 0.05 a	nd	10.6 ± 0.35 b	GC, MS
33	α-Terpineol	1190	4.2 ± 0.2 a	5.8 ± 0.85 a	6.9 ± 0.35 a	GC, MS
34	Myrtenol	1194	1.3 ± 0.2 a	nd	2.4 ± 0.00 b	GC, MS
35	(*Z*)-Piperitol	1195	nd	1 ± 0.1 a	0.2 ± 0.00 b	GC, MS
36	Dihydrocarvone	1197	1.4 ± 0.2	nd	nd	GC, MS
37	(*E*)-Isopiperitenol	1199	nd	0.7 ± 0.00 a	1.9 ± 0.25 b	GC, MS
38	(*E*)-Piperitol	1207	1.6 ± 0.00 a	1.4 ± 0.05 a	0.4 ± 0.05 b	GC, MS
39	(*Z*)-Isopiperitenol	1218	nd	nd	1.6 ± 0.65	GC, MS
40	(*E*)-Carveol	1220	7.3 ± 0.2 a	3.6 ± 0.00 b	4.2 ± 0.3 b	GC, MS
41	(*Z*)-Carveol	1232	3.7 ± 0.1 a	1.00 ± 0.00 b	11.4 ± 0.4 c	GC, MS
42	Carvone	1244	4.95 ± 0.05 a	0.5 ± 0.00 b	3.7 ± 0.15 c	GC, MS
43	Carvotanacetone	1251	0.4 ± 0.1	nd	nd	GC, MS
44	(*Z*)-Chrysanthenyl acetate	1256	nd	0.2 ± 0.05 a	0.1 ± 0.05 a	GC, MS
45	Ionene	1257	0.25 ± 0.15	nd	nd	GC, MS
46	Geraniol	1258	nd	nd	1.2 ± 0.45	GC, MS
47	Perilla aldehyde	1273	nd	nd	0.2 ± 0.00	GC, MS
48	Isobornyl acetate	1285	nd	nd	0.1 ± 0.00	GC, MS
49	*p*-Cymen-7-ol	1290	1.25 ± 0.05 a	0.8 ± 0.05 b	nd	GC, MS
50	Perilla alcohol	1290	nd	0.2 ± 0.00 a	1.3 ± 0.15 b	GC, MS
51	*p*-Menth-1-en-9-ol	1296	nd	nd	0.5 ± 0.00	GC, MS
52	Carvacrol	1299	0.4 ± 0.00	nd	nd	GC, MS
53	(*E*)-Cinnamyl alcohol	1303	0.25 ± 0.05	nd	nd	GC, MS
54	*p*-Mentha-1,4-dien-7-ol	1330	nd	0.9 ± 0.00 a	0.3 ± 0.15 a	GC, MS
55	Myrtenyl acetate	1334	0.95 ± 0.15	nd	nd	GC, MS
56	*β*-Damascenone	1384	1.7 ± 0.1 a	0.3 ± 0.05 b	nd	GC, MS
57	Geranyl acetate	1385	nd	nd	0.2 ± 0.05	GC, MS
58	10-(Acetylmethyl)-3-carene	1387	8.6 ± 0.5	nd	nd	GC, MS
59	(*E*)-*ß*-ionone	1389	0.2 ± 0.00	nd	nd	GC, MS
60	Spathulenol	1578	0.95 ± 0.75 a	8.3 ± 0.9 b	1.8 ± 0.1 a	GC, MS
61	Dillapiol	1626	0.2 ± 0.00 a	0.5 ± 0.1 a	0.3 ± 0.00 a	GC, MS
62	Isospathulenol	1640	nd	2 ± 0.25	nd	GC, MS
63	α-Bisabolol	1684	nd	0.4 ± 0.05 a	1.1 ± 0.45 a	GC, MS
*Aldehydes*			1.2	1.6	0.3	
*Ketones*			0.4	0	0	
*Esters*			0.25	0	0	
*Phenols*			0.4	0	0	
*Phenylpropanoids*			0.2	0.5	0.3	
*Monoterpene hydrocarbons*			12.55	3.4	4.2	
*Monoterpenoids*			75.25	77.3	87.4	
*Sesquiterpenes*			0	0	0.6	
*Sesquiterpenoid hydrocarbons*			0.95	10.7	2.9	
**Total chromatogram identified (%)**			91.2	93.5	95.7	

LRI—linear retention indices, nd—not detected. Identified compounds are expressed as mean ± SD. Different letters (a–c) denote statistically significant difference (*p* < 0.05).

**Table 3:** Antioxidant activity and inhibition of acetyl- (AChE) and butyryl- (BuChE) cholinesterase by hydrolates from different sea fennel plant parts.

Plant Part	Antioxidant Activity			Enzyme Inhibition	
	FRAP	DPPH	ORAC	AChE	BuChE
	(mM Fe^2+^/L)	(Inhibition %)	(µmTE/L)	(Inhibition %)	(Inhibition %)
**Leaves**	n.a.	6.30 ± 1.48	122.98 ± 0.50	n.a.	4.59 ± 2.56
**Flowers**	n.a.	6.11 ± 1.68	103.22 ± 0.44	5.05 ± 0.75	3.04 ± 1.73
**Fruits**	n.a.	5.67 ± 0.38	98.88 ± 1.00	n.a.	n.a.

n.a.—not active, detected. All results are expressed as mean ± SD.

**Table 4:** Results of linear regression for the cytotoxicity of sea fennel leaf, flower, and fruit essential oils on cancer (MDA-MB-231, MCF-7, and OVCAR-3) cell lines.

Cell Line	Predictor	Coefficient	Standard Error	F-Value	R^2^	*p*-Value
**MCF-7**	Concentration (µg/mL)	−0.226	0.03			<0.0001
	Octanal	75.86	13.01			<0.0001
	Intercept	91.10	5.153	48.00	0.627	<0.0001
**MDA-MB-232**	Concentration (µg/mL)	−0.17	0.03			<0.0001
	Octanal	74.10	11.81			<0.0001
	Time (h)	−0.43	0.09			<0.0001
	Intercept	110.66	5.83	33.00	0.645	<0.0001
**OVCAR-3**	Concentration (µg/mL)	−0.064	0.016			<0.0001
	Dodecane	36.695	7.174			<0.0001
	Intercept	85.833	3.018	21.56	0.481	<0.0001

**Table 5:** Results of linear regression for the cytotoxicity of sea fennel leaf, flower, and fruit hydrolates on cancer (MDA-MB-231, MCF-7, and OVCAR-3) cell lines.

Cell Line	Predictor	Coefficient	Standard Error	F-Value	R^2^	*p*-Value
**MCF-7**	Time (h)	−0.28	0.11			0.025
	Intercept	96.48	4.853	6.92	0.409	<0.0001
**MDA-MB-232**	Time (h)	−1.51	0.17			<0.0001
	Intercept	137.88	7.54	80.93	0.89	<0.0001
**OVCAR-3**	Time (h)	−0.254	0.085			0.014
	Intercept	77.477	3.835	8.83	0.47	<0.0001

## Data Availability

The original contributions presented in this study are included in the article/Supplementary Material; further inquiries can be directed to the corresponding author/s.

## Supplementary Materials

The following supporting information can be downloaded at: https://www.mdpi.com/article/10.3390/biom15050666/s1, Figure S1: Total ion chromatogram of sea fennel essential oils from (a) leaves, (b) flowers, and (c) fruits; Figure S2: Total ion chromatogram of sea fennel hydrolates from (a) leaves, (b) flowers, and (c) fruits; Figure S3: Percentage of metabolically active human embryonic kidney HEK-293 cell line after 24, 48 and 72 h of incubation with different concentrations of sea fennel flower and fruit essential oils; Table S1: Results of linear regression for the cytotoxicity of sea fennel flower and fruit essential oils on healthy HEK-293 cell line.

## Abbreviations

The following abbreviations are used in this manuscript:

EO	essential oil
HD	hydrodistillation
VOC	volatile organic compound
GC-MS	gas chromatography–mass spectrometry
HS-SPME	headspace solid-phase microextraction
FRAP	ferric-reducing antioxidant power
DPPH	2,2-diphenyl-1-picrylhydrazyl
ORAC	oxygen radical absorbance capacity assay
AChE	acetylcholine
BuChE	butyrylcholine
MDA-MB-231	human breast adenocarcinoma
MCF-7	human breast metastatic adenocarcinoma
OVCAR-3	human ovarian carcinoma
HER-293	healthy human embryonic kidney
ANOVA	analysis of variance
