# Supplementary material for: Biological Activities of Essential Oils and Hydrolates from Different Parts of Croatian Sea Fennel (Crithmum maritimum L.)
Source: Biomolecules. 2025 May 4;15(5):666. doi: 10.3390/biom15050666 (PMC12108800; doi:10.3390/biom15050666)
Supplement: Supplementary file 1 [file biomolecules-15-00666-s001.zip › Suppl. Figures.pdf]

biomolecules

MDPI

Article

~~What smells so good?~~ Biological activities of essential oils and hydrolates from different parts of Croatian sea fennel (*Crithmum maritimum* L.)

Livija Slišković[1], Nikolina Režić Mužinić[2], Olivera Politeo[3], Petra Brzović[4], Josip Tomaš[4], Ivana Generalić Mekinić[4,*], Marijana Popović[5,*]

a)

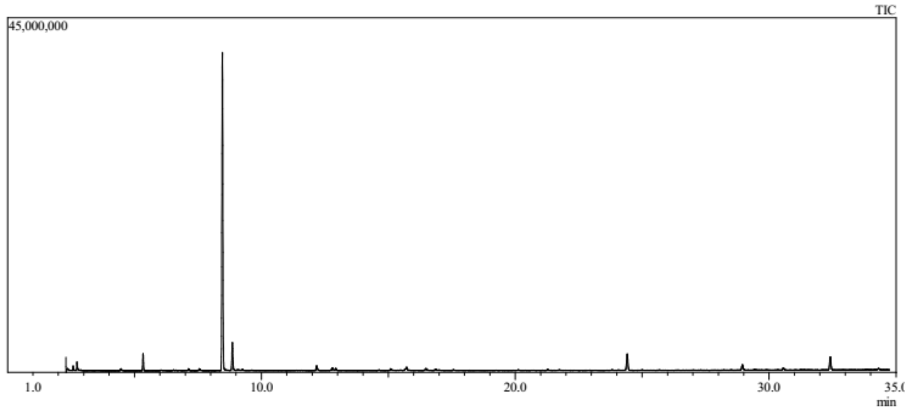

b)

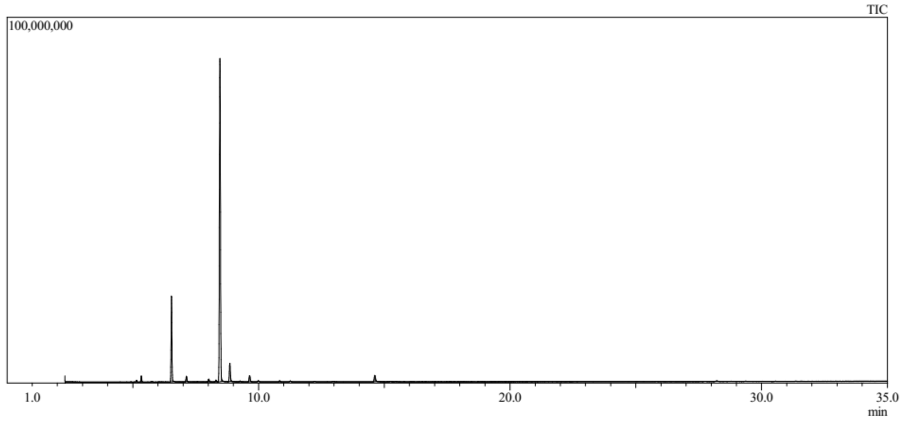

c)

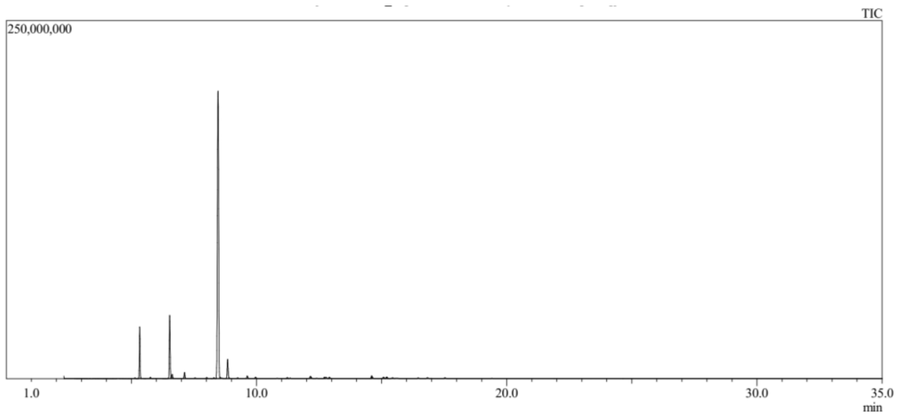

Figure S1: Total ion chromatogram of sea fennel essential oils from (a) leaves (b) flowers and (c) fruits

(a)

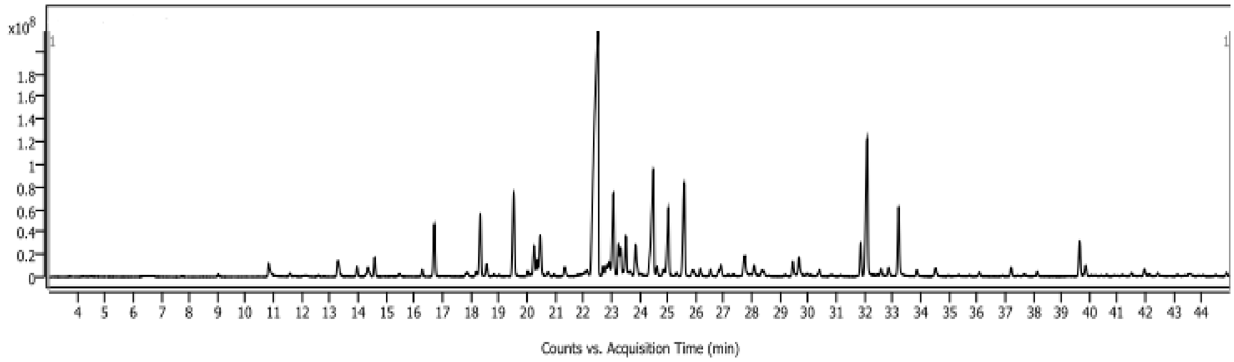

(b)

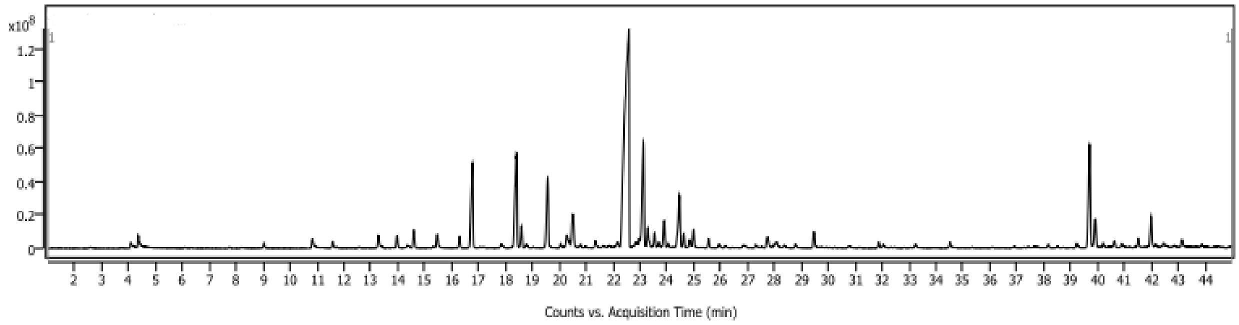

(c)

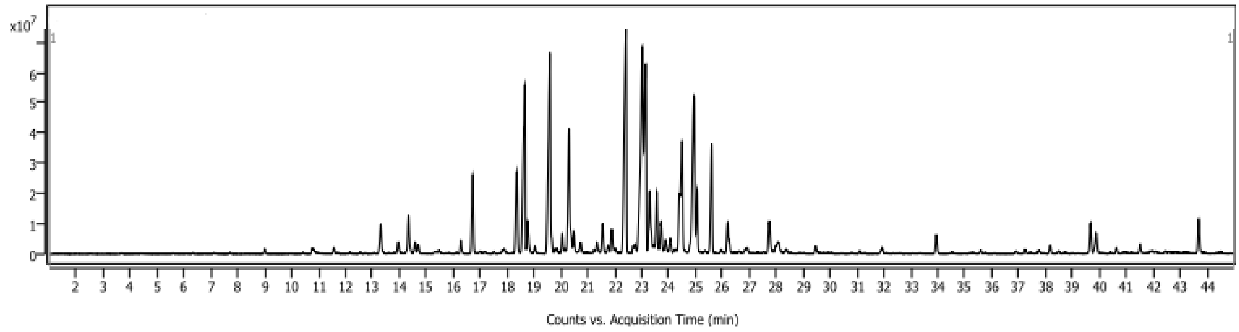

Figure S2: Total ion chromatogram of sea fennel hydrolates from (a) leaves (b) flowers and (c) fruits

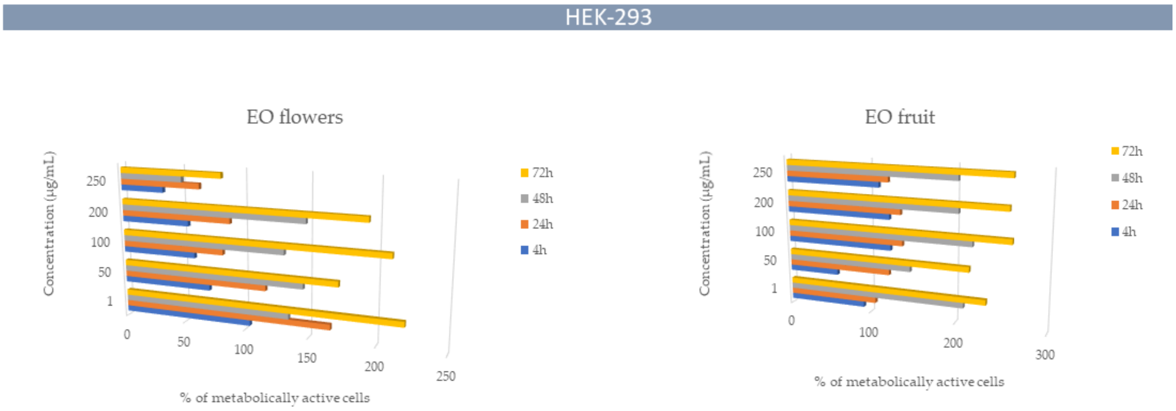

Figure S3: Percentage of metabolically active human embryonic kidney HEK-293 cell line after 24, 48 and 72 h of incubation with different concentrations of sea fennel flower and fruit essential oils
