# Supplementary material for: Biological Activities of Essential Oils and Hydrolates from Different Parts of Croatian Sea Fennel (Crithmum maritimum L.)
Source: Biomolecules. 2025 May 4;15(5):666. doi: 10.3390/biom15050666 (PMC12108800; doi:10.3390/biom15050666)
Supplement: Supplementary file 1 [file biomolecules-15-00666-s001.zip › Suppl. Tables.pdf]

biomolecules

MDPI

Article

~~What smells so good?~~ Biological activities of essential oils and hydrolates from different parts of Croatian sea fennel (*Crithmum maritimum* L.)

Livija Slišković[1], Nikolina Režić Mužinić[2], Olivera Politeo[3], Petra Brzović[4], Josip Tomaš[4], Ivana Generalić Mekinić[4,*], Marijana Popović[5,*]

Table S1. Results of linear regression for the cytotoxicity of sea fennel flower and fruit essential oils on healthy HEK 293 cell line

**HEK 293**	**Concentration (µg/mL)**	-0.094	0.056			0.09
	**Tetradecane**	-206.74	41.96			< 0.0001
	**Time (h)**	1.868	0.202			< 0.0001
	Intercept	109.63	12.39	37.61	0.758	< 0.0001
